# Benefits of Selenium Supplementation on Leukocyte DNA Integrity Interact with Dietary Micronutrients: A Short Communication

**DOI:** 10.3390/nu8050249

**Published:** 2016-04-27

**Authors:** Nishi Karunasinghe, Shuotun Zhu, Lynnette R. Ferguson

**Affiliations:** 1 Auckland Cancer Society Research Centre, Faculty of Medical and Health Sciences, The University of Auckland, Private Bag 92019, Auckland 1142, New Zealand; l.ferguson@auckland.ac.nz; 2 Discipline of Nutrition, Faculty of Medical and Health Sciences, The University of Auckland, Private Bag 92019, Auckland 1142, New Zealand; st.zhu@auckland.ac.nz

**Keywords:** Se supplementation, DNA damage, dietary folate, dietary methionine, GPx activity, caspase-cleaved keratin 18

## Abstract

A male cohort from New Zealand has previously shown variability in Selenium (Se) supplementation effects on measured biomarkers. The current analysis is to understand the reasons for variability of the H_2_O_2_-induced DNA damage recorded after Se supplementation. We have looked at the variation of demographic, lifestyle, medication, genetic and dietary factors and biomarkers measured at baseline and post-supplementation in these two extreme subgroups A and B. Group A showed increased H_2_O_2_-induced DNA damage and group B showed decreased damage after Se supplementation. We have also considered correlations of biomarkers and dietary factors in the complete dataset. The glutathione peroxidase (GPx) activity and DNA damage were significantly lower at post-supplementation in Group B compared to Group A. Post-supplementation, Group B showed a significant reduction in the GPx activity, while Group A showed a significant increase in DNA damage compared to baseline levels. Dietary methionine intake was significantly higher and folate intake was significantly lower in Group B compared to Group A. Se supplementation significantly increased the caspase-cleaved keratin 18 levels in both groups, indicating increased apoptotic potential of this supplement. Parameter correlation with the complete dataset showed dietary methionine to have a significant negative correlation with H_2_O_2_-induced DNA damage post-supplementation. The data suggest that Se supplementation is beneficial for the leukocyte DNA integrity only in interaction with the dietary methionine and folate intake.

## 1. Introduction

The beneficial effect of Se supplementation for men’s health is still a controversial issue [1,2,3,4]. Our Se supplementation (200 μg/day as selenized yeast for six months) studies from Auckland, New Zealand indicated that beneficial effects of Se vary considerably among individuals. Using baseline and post-supplementation related biomarker changes of this group; we have reported that benefits of Se are influenced by demographic, lifestyle and health factors, as well as with gene polymorphisms in the seleno-genome [5,6,7]. Our studies also indicated that Se supplementation increased the H_2_O_2_-induced DNA damage in 56.6% of New Zealand men while the erythrocyte thioredoxin reductase (TR) activity was increased in 60% of this population [5,6]. Cassidy *et al.* have shown that the electrophile bound TR can disrupt p53 confirmation leading to caspase activation [8]. Therefore, it is interesting to know whether increased TR activity and the H_2_O_2_-induced DNA damage observed with Se supplementation have any association with the homeostatic caspase-cleaved apoptosis levels in these men.

Apoptosis is proposed to be part of the beneficial effects of Se supplementation [9] and is a parameter worth assessing in Se supplementation studies. Among the main cytoskeletal proteins in epithelial cells is the cytokeratine 18 (CK18) [10]. Various caspases cleave intermediate filaments of these proteins during apoptosis. Cleavage of CK18 is said to occur at aspartic acid residue 396 [11] and is detectable with the M30 monoclonal antibody [11]. This cleavage is initiated by caspase 9 and followed by the action of caspases 3 and 7 [12].

Imbalances in protein and carbohydrate consumption have also been shown to affect DNA fragmentation in rat models [13]. The influence of saturated and polyunsaturated fatty acid dietary intakes with DNA adduct formation has also been reported before [14,15,16]. Meanwhile, the mechanism of action on DNA integrity related to Se is reportedly influenced by other micro-nutrient co-factors, including Vitamin B6, B12, zinc, folate, methionine, betaine and choline [17,18,19,20].

We present here an initial hypothesis-generating analysis to identify possible reasons associated with the extreme increases and decreases of H_2_O_2_-induced DNA damage recorded from peripheral blood leukocytes of men after six months of Se supplementation. Data reviewed include demographic, lifestyle and medication factors, biomarkers and the single nucleotide polymorphisms in antioxidant genotypes between these subgroups [6,7]. Additionally, we assess the influence of dietary and activity levels and caspase-cleaved K18 (CCK18) levels measured in plasma between these extreme subgroups. Testing of the hypothesis generated was carried out using correlation between biomarkers and dietary intake in a complete dataset.

## 2. Material and Methods

### 2.1. Sample Selection and Previous Data Access

A total of 572 men took part in a Se supplementation study protocol for six months with 200 μg/day Se given as selenized yeast (SelPlex from Alltech, Dunboyne, Ireland), (ethics reference NTY/06/07/060). Two batches of SelPlex supplements were used in this supplementation protocol, batch number 021106 (expiry November 2008) and batch number 010807 (expiry August 2009). Of those original subjects, 480 (84.5%) participants completed the study protocol [6]. A subgroup of nineteen men that showed the highest increase in H_2_O_2_-induced DNA damage (Group A) and another nineteen men that showed the highest decrease in H_2_O_2_-induced DNA damage (Group B) since Se supplementation were selected for the hypotheses-generating part of this analysis. Biomarkers including serum Se, GPx and thioredoxin reductase (TR) activity level measured from erythrocyte lysates, leukocyte DNA damage quantitated by Comet assay as % tail DNA with and without a 4 °C H_2_O_2_ challenge, demographic, lifestyle, medication factors as well as seleno-genotype data were accessed for these men from our previous studies [5,6]. Biomarker data from a total of 362 men that completed the Se supplementation protocol [5,6] and have also provided food and activity diaries were selected for the hypothesis testing part of this study.

### 2.2. Dietary and Activity Level Assessment

At study recruitment, participants were provided with a four day food and activity diary with guidelines for record completion (Supplementary document). Completed diaries were collected at the baseline time point. Data collected were analysed using the FoodWorks Professional (Version 7, Xyris software Australia Pty Ltd., Brisbane, Australia). Among the data extracted for the initial part of the study were the % energy contribution by carbohydrates, proteins and fats, % of fats as saturated, poly-unsaturated and mono-unsaturated components, and micro-nutrient components of Se, Zinc, Vitamin B6, B12, folate and methionine. The activity level recorded by these men between seven strata as used in the FoodWorks data analysis system was used to estimate the activity level variation in these men.

### 2.3. Measurement of CCK18 Levels in Subgroups

Heparin plasma collected from selected men at baseline and post-supplementation time points and stored at −80 °C since 2006–2009 were used for the analysis of CCK18 levels in October 2014. Plasma samples were thawed in a 37 °C water-bath for approximately 5 min and immediately transferred on to ice. Samples were vortexed and also inverted 10 times to secure adequate mixing. The measurements of CCK18 levels were carried out using two M30 Apoptosense ELISA solid-phase sandwich enzyme immunoassay kits, from Peviva, VLVbio, Sundbyberg, Sweden, using the manufacturer’s protocol. Each kit was able to analyse 19 baseline and post-Se supplemented samples in duplicate. 3,3′,5,5′-Tetramethylbenzidine bound horseradish peroxidase conjugated M30 antibody was measured at 450 nm using a Spectramax M2 plate reader from Molecular Devices, Sunnyvale, CA, USA. The absorbance data of standards from these kits were fitted to Four Parameter Logistic Curves (*R*^2^ = 0.9993 and 0.9998) using Sigma plot 11.0 and the predicted analyte concentrations for test samples were derived against absorbance measurements.

### 2.4. Statistical Analysis

Categorical variables between Groups A and B were described as frequency and percentage. These variables were compared between the two groups using the Fisher exact test. Continuous variables that were not normally distributed were analysed between Groups A and B using the Mann-Whitney Rank Sum Test. These variables were described as the median and 25th and 75th percentiles. Variations between baseline and post-supplementation data for these variables were compared using the Wilcoxon signed rank test with pooled ranks from both time points. The normally distributed continuous variables were compared with the Student’s *t*-test between Groups A and B or using paired *t*-test between baseline and post-supplementation time points. These variables were described as mean and standard deviation. *p*-values < 0.05 were considered significant. As this is a hypothesis generating study, correction for multiple testing was not carried out.

Biomarkers and the dietary folate and methionine intakes from a total of 362 men were tested using the Spearman’s Rank Order Correlation for both baseline and post-supplementation time points. *p*-values < 0.05 were considered significant.

## 3. Results

### 3.1. Comparison of Subgroup Characteristics

The characteristics of men selected for Groups A and B are given in Table 1. Demographic, lifestyle, activity level, medication taken and genotype data between the two groups showed no significant difference. However, the mean age was 6.4 y lower in Group B compared to Group A. Group A also showed a non-significantly higher intake of cardiovascular medication compared to Group B (47.37% *vs.* 21.05%). The proportion of the GPx1 rs1050450 minor allele *T* was 34.21% and 16.67% respectively between Groups A and B, although this difference was not statistically significant. The proportion of the *SEPP1* rs3877899 minor allele *A* was 31.58% and 16.67% respectively between Groups A and B although this also was not statistically significant.

### 3.2. Comparison of Subgroup Variability in Nutrient Intake

Dietary intake variation between Groups A and B is given in Table 2. None of the macro-nutrient intakes were different between the groups. Except for the intake of folate and methionine levels, the rest of the micronutrients assessed also showed no significant difference between the two groups. Group B shows 32% less consumption of total folates (median 348.49, 25th and 75th percentiles 298.98 and 461.74 respectively) compared to Group A (median 504.79, 25th and 75th percentiles 370.10 and 616.30 respectively), (*p* = 0.046). Group B shows 45% more consumption of methionine compared to Group A (mean of 1.09 ± 0.44 *vs.* 0.75 ± 0.37, *p* = 0.039).

### 3.3. Comparison of Subgroup Variability in Previously Assessed Biomarkers

**Figure 1:**
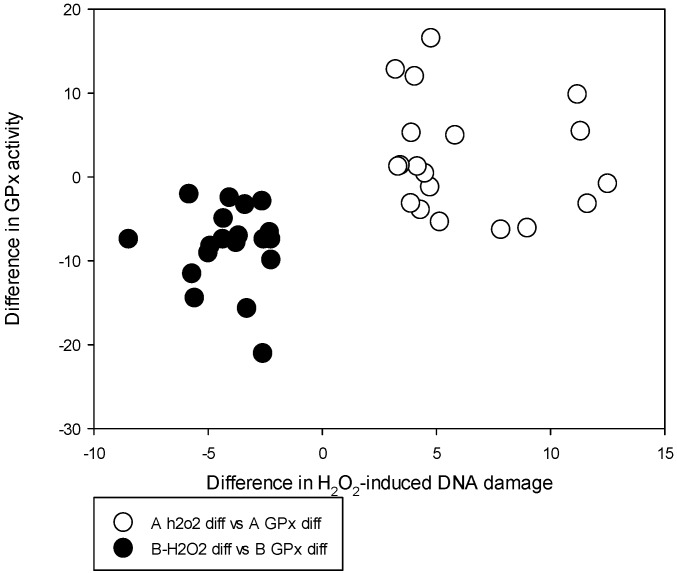
Distribution of individual differences of the GPx activity levels against difference in the H_2_O_2_-induced DNA damage levels among Groups A and B between baseline and post-supplemented time points.

Biomarker variations between the two groups at baseline and post-supplementation time points are given in Table 3. The serum Se levels at both baseline and post-supplementation time points showed no statistically significant difference between Groups A and B. Supplementation has increased the serum Se levels significantly in both groups as expected. There were no significant differences in the GPx activity at baseline or the TR activity at both time points in both groups. However, post-supplementation levels of the GPx activity show a difference, with Group B recording a significantly lower level as compared to Group A (9.65 ± 4.92 *vs.* 19.10 ± 6.03, *p* < 0.001). Group B also shows a significant reduction in the GPx activity with Se supplementation compared to their baseline level 18.58 (25th and 75th percentiles 15.69 and 21.47 respectively) and all individuals in Group B have shown this reduction post-supplementation (Figure 1). At baseline there was no significant difference in the % tail DNA in fresh blood leukocytes between groups A and B. Group A showed a significant increase from 5.40 (25th and 75th percentiles 4.83 and 6.70) to 6.93 (25th and 75th percentiles 5.90 and 12.74) in the post-supplemented % tail DNA in fresh blood leukocytes (*p* < 0.03). Group B showed a significant decrease from 6.04 (25th and 75th percentiles 5.40 and 6.65) to 5.45 (25th and 75th percentiles 4.90 and 6.06) in the post-supplemented % tail DNA in fresh blood leukocytes (*p* < 0.04). Group B also recorded a significantly lower level of the % tail DNA in fresh blood leukocytes compared to Group A at post-supplementation time point 5.45 (25th and 75th percentiles 4.90 and 6.06) compared to 6.93 (25th and 75th percentiles 5.90 and 12.74), *p* = 0.002.

### 3.4. Comparison of Subgroup Variability in CCK18 Levels

**Figure 2:**
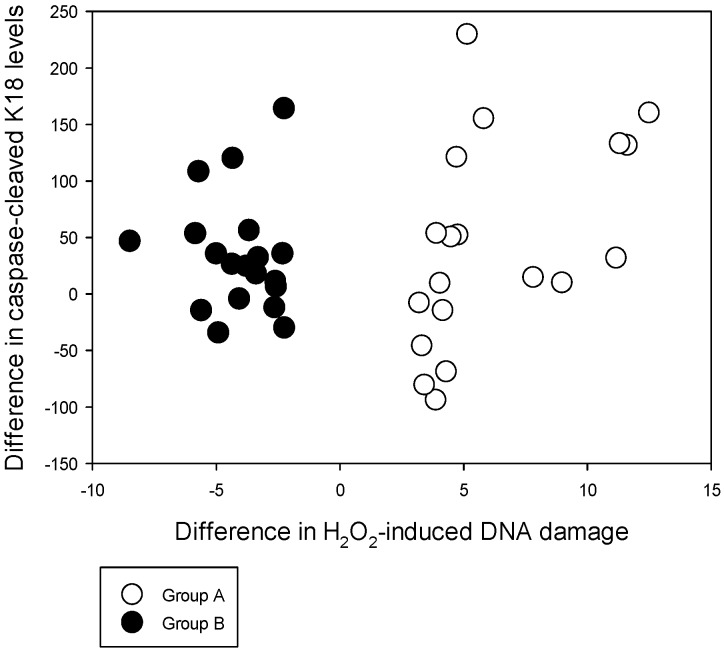
Distribution of individual differences of the caspase-cleaved K18 levels against differences in the H_2_O_2_-induced DNA damage levels among subgroups A and B between baseline and post-supplemented time points.

The intra-assay coefficient of variability (CV) of the CCK18 levels varied between 0.07% and 5.25% while the inter-assay variability varied from 9.15% to 11.44% in assay standards. Three test samples that had %CV beyond 20 were removed from the analysis. The CCK18 levels between the two groups at baseline and post-supplementation time points are given in Table 4. The CCK18 level did not differ between Groups A and B at either time point although Group B showed a non-significant increase particularly at baseline. However, both groups had significant increases of the CCK18 levels post-supplementation compared to their baseline levels. Individual variation in the difference of this analyte between baseline and post-supplementation time points also indicates that the majority in both groups have an increase of the CCK18 with Se supplementation (Figure 2).

### 3.5. Correlation between Parameters of the Complete Dataset

#### 3.5.1. Baseline

Complete data at baseline showed a significant positive correlation between serum Se and the seleno-enzyme activities in erythrocyte lysates (GPx activity and serum Se-Correlation coefficient = 0.186, *p* < 0.001, TR activity and serum Se-Correlation coefficient = 0.134, *p* = 0.011). GPx activity showed a significant positive correlation with H_2_O_2_-induced DNA damage (Correlation coefficient = 0. 0.109, *p* = 0.038). Correlations were also observed between the GPx and TR activities (Correlation coefficient = 0.180, *p* < 0.001) and % H_2_O_2_-induced tail DNA and % fresh blood tail DNA (Correlation coefficient 0.207, *p* < 0.001). Data also showed a statistically non-significant trend towards a negative correlation between dietary folate intake and both % fresh blood tail DNA and the GPx activity (Table 5).

#### 3.5.2. Post-Supplementation

At post-supplementation, dietary methionine intake showed a negative correlation with H_2_O_2_-induced DNA damage (Correlation coefficient = −0.109, *p* = 0.039) (Table 6). Correlations seen at baseline between serum Se and seleno-enzymes, GPx and TR activities and fresh blood and H_2_O_2_-induced DNA damage were not seen at post-supplementation. Correlation between GPx activity and H_2_O_2_-induced DNA damage remains similar to baseline (Correlation coefficient = 0.104, *p* = 0.049). Additionally, correlation was seen between GPx activity and fresh blood DNA damage at post supplementation (Correlation coefficient = 0.107 and *p* = 0.042).

## 4. Discussion

Our current analysis shows that the extremes of the H_2_O_2_-induced DNA damage are not associated with the demographic, lifestyle, and medication factors. There were no significant variations among the % energy contribution from major nutrients or % of fats as saturated, poly-unsaturated and mono-unsaturated components, or intake of Se, Vitamin B12, Vitamin B6 or zinc. However, dietary components of folate and methionine were marginally but significantly different between the two groups. These two nutrients are known for their one carbon metabolism that provides methyl groups for various methyl transfer reactions [21]. Methionine is an essential amino acid providing methyl groups for methylation of bio-molecules including nucleic acids [22].The process of methyl transfer is co-supported by folate, and both are ubiquitously found in all living cells [21]. Therefore, any imbalance in these nutrients can have a profound influence on DNA integrity and methylation reactions [21]. The requirement of folate in maintaining DNA stability is thought to be due to its ability to prevent excessive uracil incorporation to DNA [23,24]. Group B records a lower dietary intake of folate compared to the recommendation of 400 μg/day specified by the Australian National Health and Medical Research Council and the New Zealand Ministry of Health (ANHMRC and NZMOH), while Group A records a level above this requirement [25]. However, there was no indication of a correlation between dietary folate intake and H_2_O_2_-induced DNA damage with the complete dataset at both baseline and post-supplementation time points. Correlation between fresh blood DNA damage and dietary folate intake for the complete dataset at baseline however showed a non-significant negative trend, which is lost after Se supplementation. Dietary methionine, however, showed significant negative correlation with H_2_O_2_-induced DNA damage post-supplementation with the complete dataset.

The ANHMRC and NZMOH have not specified a methionine nutritional requirement on its own, although they have discussed the influence of other nutrients including cysteine and folate in maintaining methionine levels [25]. According to the World Health Organisation standards, dietary requirement of the sulphur amino acid methionine on its own is 10.4 mg·kg^−1^·day^−1^ [26]. The median body weight of Group A was 78 kg which gives an estimated methionine requirement of 0.81 g per day. The median body weight of Group B was 75 kg which gives an estimated methionine requirement of 0.78 g per day. Group A recorded a dietary methionine level 7.4% below the estimated requirement, while Group B recorded a level 39.7% above the estimated requirement. Methionine supplemented diets have previously shown 122% increase of micronuclei frequency in peripheral blood without any increases of these marker in heart and liver tissue of adult female mice [27]. Rodents supplemented with 3 fold higher L-methionine levels compared to controls have shown mitochondrial ROS generation and oxidative damage to mitochondrial DNA in liver tissue [28]. Meanwhile, a 40% methionine restricted diet has shown a decrease in mitochondrial ROS production, reduced damage in mitochondrial DNA and decreased methylation in genomic DNA in rat liver tissue [29,30]. As methionine is the ultimate source of methyl groups in DNA methylation reactions, it is known that dietary methionine intake can control methylation reactions in mammals [31]. Waterland has also reviewed methionine supplementation effects on S-adenosylmethionine (SAM) and S-adenosylhomocysteine (SAH), the latter of which is converted from the former by the DNA methyltransferase reactions. According to Waterland, there exists a complex variation in ultimate SAM to SAH ratios based on the age of animals as well as based on other nutrient co-factors provided through the supplemented diet. Our subgroup data suggests that men in Group B have overcome folate deficiency and methionine excess by supplemented Se, while those with sufficient levels of folate and relatively lower levels of methionine showed detrimental effects from Se supplementation. Ingested selenomethionine (SeMet) can either get directly incorporated to proteins instead of methionine or metabolised to other low molecular weight selenium containing molecules such as selenohomocysteine and selenocysteine. The latter happens through a methionine cycle and a trans-sulfuration pathway as described by Lazard *et al.* [32]. Lazard *et al.* have further confirmed production of superoxide radicals by SeMet treatment in mutant yeast strains. They also discuss ready oxidation of low molecular weight metabolites of SeMet such as selenocysteine and selenohomocysteine as the basis of superoxide radical formation, rather than being direct effects of SeMet. The organic Se supplement SelPlex has approximately 34%–36% of low molecular weight seleno molecules apart from having 63% of SeMet [33,34]. These low molecular weight seleno molecules too could have the potential of getting oxidised to form superoxide and other free radicals. Increased H_2_O_2_-induced DNA damage displayed by individuals in Group A after Se supplementation could be due to such free radical effects of SelPlex metabolites although the majority of participants from the complete study cohort have not recorded symptomatic toxicities [6]. However, higher methionine levels in Group B individuals seem to have supressed SeMet induced free radical formation and subsequent DNA damage. Meanwhile, it has been reported that the ribosomes fail to distinguish between methionine and SeMet loaded tRNA during the process of translation [35,36]. This makes it possible for SeMet to modulate the effects of excess methionine by competing for its translation into proteins. On the other hand, Lazard *et al.* have recorded the efficacy of methionine (0–40 μM) in suppressing SeMet (2.5–20.0 μm) related growth impacts in *Saccharomyces cerevisiae* [32]. It may well be that both methionine and SeMet can modulate damaging effects of one another. If folate is sufficiently available, that could support recycling of methionine from homocysteine or SeMet from selenohomocysteine using folate dependent methionine synthase [32,37]. This could reduce the chances of free radical formation in the process of homocysteine or selenohomocysteine oxidation. Therefore, low folate levels in Group B have shown higher DNA damage at baseline as recycling of endogenous homocysteine and selenohomocysteine would have been impaired.

The significant decrease in the GPx activity corresponding to decreased H_2_O_2_-induced DNA damage post-supplementation in Group B is similar to our previous recording of supplementation effects shown by 141 men from the highest baseline tertile of H_2_O_2_-induced DNA damage [6]. The increase in the fresh blood DNA damage among Group A and a decrease in Group B after supplementation are similar to supplementation variation recorded with the highest and lowest baseline fresh blood DNA damage tertiles recorded from 424 men from our previous studies [6]. These data indicate that the biomarker data of this small group of 38 men are representative of the dataset from our original cohort of men that showed a variation of DNA damage with Se supplementation. A review of known GPxs by Brigelius-Flohe and Maiorino records that the phospholipid hydroperoxide glutathione peroxidase (GPx4) is an important modulator of apoptosis [38]. In particular, the 12, 15 lipoxygenase (LOX)-induced apoptosis caused by activation of the apoptosis inducing factor (AIF) instead of caspase activation is shown to be modulated by GPx4 [38]. Previous studies indicate that, depending on Se availability, the function of different GPxs can interchange their leading roles Using chimeric constructs having mutually exchanged 3′UTR regions in GPx1, GPx4 and GPx2 coding regions, it has been shown that both GPx4 and GPx1 activities can get overexpressed under Se adequate conditions but not the GPx2 [39]. Our GPx activity records were from erythrocyte lysates which measure only the cytosolic GPx1 activity, and we cannot prove that the reduction of GPx activity in Group B is compensated by activation of GPx4 and GPx2.

Men in both extreme subgroups show a significant (~25%) increase in the CCK18 levels with Se supplementation suggesting an increase of homeostatic caspase-induced apoptosis. It has been proposed that health benefits of Se could well be due to its apoptotic effects [9,40]. Effects of Se on apoptosis is recorded in *in vitro* experiments with various cell lines including SWO-38 human glioma cells, A549/DDP human lung adenocarcinoma cells, HL-60 leukaemia cell, a549 human lung cancer cells, MCF-7 human breast adenocarcinoma cells, normal human skin fibroblasts, cervico-uterine cancer cells and prostate cancer cells. These experiments have used a variety of inorganic and organic Se supplements including sodium selenite, SeMet, modified Se nanoparticles, methylseleninic acid, Se-containing phycocyanin (Se-PC) and 22-oxo-26-selenocyanocholestane [41,42,43,44,45,46,47,48,49]. Hawkes *et al.* have carried out a Se supplementation study with 16 healthy men for 1 year with 300 μg/day of high selenized yeast or placebo. Whole blood gene expression with DNA micro-array data from these men before and after Se supplementation has shown that the main functional pathway affected by Se supplementation was that of FAS apoptosis signalling [50]. The magnitude of CCK18 increase in our cohort is comparable with such increases recorded after Docetaxal treatment on hormone refractory prostate cancer patients (~20% CCK18 level increase) [51]. With cultured Synoviocytes, it has been shown that the caspases-8 and 9 (apoptosis initiator caspases) and caspase-3 (apoptosis executioner caspase) are upregulated in folate-deprived conditions compared to folate sufficient conditions [52]. These authors have further shown that folate deprivation was associated with a two fold increase in reactive oxygen species (ROS) compared to that of the folate supplemented medium. Using a rat model treated with the chemotherapeutic agent Cisplatin, Bodiga *et al.* have shown that those supplemented with folate show an attenuation of Cisplatin induced apoptosis in the intestinal epithelium. They have shown this decrease using M30 staining, DNA fragmentation, and caspase-3 activity [53]. With our data, the extremes of H_2_O_2_-induced DNA damage cannot be significantly associated with variable changes in the caspase-cleaved apoptosis levels. However, those with lower dietary folate and higher dietary methionine (Group B) could have been exposed to higher caspase-cleaved apoptosis at pre-supplementation compared to Group A, which subsequently produced a non-significant increase in the CCK18 levels. Therefore, our results could be indicating that Se supplementation produces a homeostatic increase in apoptosis while modulating subsequent lower folate and higher methionine related effects on leukocyte DNA integrity.

The concentrations of Se that up-regulated the CCK18 levels in plasma in Groups A and B were within the limits recorded in Zhao *et al.* study for up regulating apoptosis [48]. Se supplementation with selenite on a cell line derived from fish has shown both apoptotic and necrotic effects beyond a level of 10 μM. Levels above 10 μM also induced mitochondrial membrane potential damage, DNA damage and elevated production of reactive oxygen species considered to be associated with cell death [54]. Using two malignant mesothelioma cell sublines, Nilsonne *et al.* have shown that apoptosis occurs between 5 and 30 μM selenite. The mean post-supplementation serum Se levels in groups A and B in our study were 177.3 ng/mL (2.25 μM/L) and 175.7 ng/mL (2.23 μM/L) respectively. These are below the 10 μM limit of Se from selenite reported in Selvaraj *et al.* [54] for fish cell lines or the levels reported from human tumour cell lines associated with apoptosis [55]. However, accumulation of Se in tissues other than in serum could have been different. Stoebe *et al.* have recorded that Se supplementation in farmed deer increased Se levels and the GPx activity in tissue at varying degrees [56]. Brozmanova *et al.* have reviewed varying mechanisms involved in apoptotic processes between different seleno-compounds [57]. Therefore, for organic Se supplements, the limit required for apoptosis could be different to that of selenite.

Although we have measured GPx activity in the erythrocyte lysates, DNA damage was measured in the leukocytes. GPx1 is the lowest in the hierarchy of known selenoproteins. Such selenoproteins are known to give way to those on top of the hierarchy (e.g., GPx4) in times of selenium deficiency [38]. As the primary outcome of our original study was to optimize Se benefits, we had to work out levels associated with optimizing the GPx1 activity. This can be measured in either erythrocyte or leukocyte lysates [58]. A study carried out with GPx activity measurements in horses reveals that levels from both erythrocytes and leukocytes are comparable among control horses, while among horses with a respiratory distress, the erythrocyte levels were higher than that of the leukocytes [58]. Erythrocyte and granulocyte GPx activity is known to be comparable in humans although lymphocytes record several fold higher levels [59]. As our DNA damage assessment protocol was with the general leukocyte population, it is represented by a higher proportion of granulocytes than lymphocytes [60]. In addition, red blood cells (RBC) have a definitive lifespan of approximately 120 days [61,62] while that of leukocytes varies [63,64]. As our supplementation time was six calendar months or approximately 180 days, we needed to assess supplementation effects in blood cells born within the supplementation period and survived through our assessment; and RBCs were the ideal candidate for that. Unfortunately, as RBCs in circulation lack a nucleus, we had no option but to select leukocytes for our DNA damage assessments.

We acknowledge several shortcomings in our study. Supplementation was carried out with two batches of SelPlex supplements, as the duration of recruitment and supplementation required a longer time than originally expected. Although SelPlex generally has 63% SeMet and 34%–36% of other low molecular weight seleno molecules, possible variation between the batches was unavoidable. We have not recorded key intermediate molecules of the methionine cycle and trans-sulfuration pathway. We have based our folate and methionine nutrition status based on dietary pattern given by a four day diet diary, which may not be an adequate measure to represent dietary intake within six months. Besides, supplementation would have changed the methionine profiles in blood. However, we do not have folate and methionine levels measured in blood at baseline and post-supplementation time points to strengthen our argument. We recommend that these molecules be assessed in future Se supplementation related studies.

## 5. Conclusions

Our extreme subgroup data showed that Se supplementation benefits for the leukocyte DNA integrity is accomplished only by those with lower dietary folate and higher dietary methionine intakes. For those with sufficient levels of folate intake and lower levels of methionine intake, Se supplementation was not favourable for the leukocyte DNA integrity. Se supplementation significantly increased the CCK18 levels at both extremes, indicating increased homeostatic apoptotic potential of this supplement. The extremes of H_2_O_2_-induced DNA damage had no significant association with the CCK18 levels, although a trend is seen among those with lower folate and higher methionine intakes recording non-significantly higher CCK18 levels. Our complete dataset reiterates the influence of supplemented Se on DNA integrity especially in the presence of high methionine intake. Future studies require stringent checking supported by assessing levels of methionine and SeMet metabolites as well as monitoring changes in nutrient profiles in blood on a periodic basis through a study duration.

## Figures and Tables

**Table 1:** Demographic, lifestyle, medication and genetic characteristics of subgroups A and B.

Demographic Data: Mean (SD) or Median and Percentiles ^1^		Group A	Group B	95% CI	*p* Value
Age (y)		59.16 (11.332)	52.79 (11.842)	−1.26 to 14.00	0.099
Weight (kg)		78 (73,90) ^1^	75 (68,88) ^1^		0.381
Height (cm)		177.55 (3.82)	175.62 (6.79)	−1.69 to 5.56	0.287
BMI		25.99 (3.80)	25.52 (3.67)	−1.98 to 2.93	0.698
Lifestyle data: N (%)				Odds ratio	*p* value
Tobacco smoker	Ever	6 (31.58)	8 (42.11)	0.642	0.737
	Never	13 (68.42)	11 (57.89)		
Alcohol intake	Yes	18 (94.74)	15 (78.95)	4.62	0.34
	No	1 (5.26)	4 (21.05)		
Activity level	>moderate	3 (23.08)	3 (18.75)	1.29	1
	<Light-moderate	10 (76.92)	13 (81.25)		
Medication N (%)	None	10 (52.63)	12 (63.16)		0.094
	Cardiovascular	9 (47.37)	4 (21.05)		
	Other	0	3 (15.79)		
Genetic polymorphisms: allele frequency N (%)					
GPx1 rs1050450	Allele C	25 (65.79)	30 (83.33)	0.389	0.112
	Allele T	13 (34.21)	6 (16.67)		
*SEPP1* rs3877899	Allele G	26 (68.42)	31 (81.58)	0.494	0.289
	Allele A	12 (31.58)	7 (16.67		

95% CI = 95% Confidence Interval; ^1^ = Median and 25th and 75th percentiles.

**Table 2:** Dietary intake recorded for subgroups A and B.

	Group A	Group B	95% CI	*p* Value
Macro nutrients: Mean (SD) or Median and percentiles ^1^				
kj-from-protein_%	15.69 (2.29)	15.54 (2.70)	−1.80 to 2.09	0.878
kj-from-carbohydrates_%	43.15 (6.60)	44.49 (8.96)	−7.45 to 4.77	0.656
kj-from-total fats_%	34.92 (30.20,42.33) ^1^	31.69 (30.64, 37.11) ^1^		0.241
fat-as-saturated_%	41.90 (8.74)	45.84 (5.51)	−9.62 to 1.74	0.26
fat-as-poly_%	20.78 (7.71)	16.54 (5.13)	−0.86 to 9.34	0.099
fat-as-mono %	37.32 (4.17)	37.62 (3.26)	−3.20 to 2.61	0.834
Micro-nutrients: Mean (SD) or Median and percentiles ^1^				
Selenium μg/day	60.15 (27.88)	59.38 (22.50)	−9.80 to 28.74	0.322
Vitamin B12 μg/day	4.18 (3.54,7.54) ^1^	5.28 (3.28,7.82) ^1^		0.629
Vitamin B6 mg/day	1.70 (1.54, 2.23) ^1^	1.81 (1.56, 2.14) ^1^		0.629
Zinc mg/day	12.39 (11.57,14.60) ^1^	15.03 (12.32, 16.14) ^1^		0.103
Total folate μg/day	504.79 (370.1,616.30) ^1^	348.49 (298.98, 461.74) ^1^		0.046
Methionine g/day	0.75 (0.37)	1.09 (0.44)	−0.65 to −0.02	0.039

^1^ = Median and 25th and 75th percentiles; kj—kilo joules.

**Table 3:** Variation in pre-assessed biomarker data between subgroups A and B and between baseline and post-supplementation time points.

		Group A	Group B	95% CI	*p* Value
Biomarker data, Mean (SD) or Median and percentiles ^1^					
Serum Se (ng/mL)	Pre-	102.65 (94.75, 126.34) ^1^	123.18 (105.02, 142.13) ^1^		0.074
	Post-	177.32 (30.95)	175.67 (24.07)	−16.58 to 19.90	0.854
		***p* ≤ 0.001**	***p* ≤ 0.001**		
GPx activity (mU/mg Hb)	Pre-	16.37 (12.49, 19.82) ^1^	18.58 (15.69, 21.47) ^1^		0.199
	Post-	19.10 (6.03)	9.65 (4.92)	5.83 to 13.07	**<0.001**
		***p* = 0.181**	***p* < 0.001**		
TR activity (mU/mg Hb)	Pre-	1.23 (0.89)	1.33 (0.73)	−0.63 to 0.44	0.708
	Post-	1.55 (0.99)	1.59 (0.89)	−0.66 to 0.58	0.893
		***p* = 0.368**	***p* = 0.358**		
% FB Tail DNA	Pre-	5.40 (4.83, 6.70) ^1^	6.04 (5.40, 6.65) ^1^		0.335
	Post-	6.93 (5.9, 12.74) ^1^	5.45 (4.90, 6.06) ^1^		**0.002**
		***p* < 0.030**	***p* < 0.040**		
% H_2_O_2_-induced Tail DNA	Pre-	7.03 (1.76)	10.02 (1.57)	−4.09 to −1.89	**<0.001**
	Post-	10.62 (9.88, 17.00) ^1^	5.94 (5.36, 6.72) ^1^		**<0.001**
		***p* < 0.001**	***p* < 0.001**		

To convert serum Se level to μM/L multiply by 0.0127; 95% CI = 95% Confidence Interval; mU = milli units; Hb-hemoglobin; FB = Fresh blood; 1U of GPx activity = 1 mmol NADPH oxidized/min at 37 °C; 1U of TR activity = 1 mmol 5-thio-2-nitrobenzoic acid formed/min at 37 °C; Pre- = Baseline; Post- = Post-supplementation; *p* values between pre- and post-supplementation comparisons are underlined; ^1^ = Median and 25th and 75th percentiles.

**Table 4:** Caspase-cleaved K18 levels in plasma [units per litre mean (SD)] between subgroups A and B and between baseline and post-supplementation time points.

	Group A	Group B	95% CI	*p* Value
Pre-	182.38 (67.5)	222.24 (70.2)	−87.215 to 7.496	0.0962
Post-	229.78 (82.5)	255.69 (79.8)	−81.790 to 29.966	0.352
	***p* = 0.0428**	***p* = 0.0218**		

Pre- = Baseline; Post- = Post-supplementation; *p* values between pre- and post-supplementation comparisons are underlined.

**Table 5:** Baseline correlations between parameters assessed for complete dataset.

	Serum Se	GPx Activity	TR Activity	% FB Tail DNA	Dietary Folate	Dietary Methionine
% H_2_O_2_-Tail DNA	0.0477	0.109	0.0758	0.207	−0.0399	0.0636
	0.367	**0.0382**	0.151	**<0.001**	0.453	0.232
	360	360	360	360	355	355
Serum Se		0.186	0.134	0.072	−0.031	−0.046
		**<0.001**	**0.011**	0.174	0.554	0.387
		362	362	360	357	357
GPx activity			0.180	0.069	−0.089	−0.034
			**<0.001**	0.19	0.0931	0.523
			362	360	357	357
TR activity				−0.0369	0.0393	−0.0214
				0.485	0.459	0.687
				360	357	357
% FB Tail DNA					−0.0897	0.0293
					0.0914	0.581
					355	355

Each cell is provided with a correlation coefficient, a *p* value estimated through the Spearman’s Rank Order Correlation and sample number tested; FB = Fresh blood.

**Table 6:** Post-supplementation correlations between parameters assessed for complete dataset.

	Serum Se	GPx Activity	TR Activity	% FB Tail DNA	Dietary Folate	Dietary Methionine
% H_2_O_2_ Tail DNA	0.079	0.104	−0.0027	0.051	0.04	−0.109
	0.134	**0.049**	0.96	0.332	0.451	**0.039**
	362	361	361	360	357	357
Serum Se		−0.0597	0.034	0.121	0.024	−0.078
		0.258	0.496	**0.021**	0.651	0.142
		361	361	360	357	357
GPx activity			−0.154	0.107	−0.054	−0.029
			0.00347	**0.042**	0.306	0.58
			361	359	356	356
TR activity				−0.029	−0.029	−0.024
				0.577	0.58	0.652
				359	356	356
% FB Tail DNA					0.019	−0.042
					0.714	0.429
					355	355

Each cell is provided with a correlation coefficient, a *p* value estimated through the Spearman’s Rank Order Correlation and sample number tested.; FB = Fresh blood.

## Supplementary Materials

The following are available online at http://www.mdpi.com/2072-6643/8/5/249/s1.

## Abbreviations

The following abbreviations are used in this manuscript:

AIF	apoptosis inducing factor
ANHMRC and NZMOH	The Australian National Health and Medical Research Council and the New Zealand Ministry of Health
CHO	Chinese Hamster ovary
CK18	Cytokeratine 18
CCK18	Caspase-cleaved K18
GPx	Glutathione peroxidase
GPx1	Glutathione peroxidase 1
GPx2	Glutathione peroxidase 2
GPx4	Glutathione peroxidase 4
H_2_O_2_	Hydrogen peroxide
LOX	Lipoxygenase
SAM	S-adenosylmethionine
SAH	S-adenosylhomocysteine
Se	Selenium
SeMet	Selenomethionine
*SEPP1*	Selenoprotein P
TR	Thioredoxin reductase
%CV	% Coefficient of variability
95% CI	95% confidence interval
