# Supplementary material for: Benefits of Selenium Supplementation on Leukocyte DNA Integrity Interact with Dietary Micronutrients: A Short Communication
Source: Nutrients. 2016 Apr 27;8(5):249. doi: 10.3390/nu8050249 (PMC4882662; doi:10.3390/nu8050249)
Supplement: Supplementary file 1 [file nutrients-08-00249-s001.zip › nutrients-121674-supple-layout final/nutrients-121674-supplementary-layout.docx]

Supplementary Materials: Benefits of Selenium Supplementation on Leukocyte DNA Integrity Interact with Dietary Micronutrients: A Short Communication

Nishi Karunasinghe, Shuotun Zhu and Lynnette R. Ferguson

| Name___________________ | CODE________________ |
| --- | --- |
| Height___________________cm | Weight___________________kg |
| DOB___________________ | Occupation___________________ |

Thank you for choosing to participate in this study. Your accurate completion of this diet survey and physical activity questionnaire will contribute towards understanding your physical well being and therefore is greatly appreciated. Please ensure that the records are kept for four consecutive days as soon as conveniently possible before your first visit to our study.

Please find details below of some simple instructions that will help you to complete the diet record most accurately.

**KEEPING AN ACCURATE DIET RECORD**

**Please read the following instructions very carefully.**

It is really important that you **do** **not** adjust what you eat and drink because you are keeping
a record.

We are only interested in your normal eating habits, not the perfect diet.

Please remember to record food **at** **the** **time** **of** **eating** and not from memory at the end of the day. This is very important for the accuracy of your record.

• Please record **all** food and drinks consumed.

• Please record the details of the cooking method (e.g., fried, grilled, boiled, roasted, steamed, poached, stewed *etc.*…).

• Try to record as many details about the food as you can. e.g., breakfast cereal (Weetbix) with milk (whole or trim).

• Include all meals and all snacks and drinks including water.

• Include any additions to the food (e.g., sauces, dressings, spreads).

• Include any dietary supplements.

RECORDING AMOUNTS

It is also important to record the quantity of the food and drink that you consume.

Here are some suggestions on simple ways to record the amounts:

1. **Use** **household** **measurements** **to** **estimate** **the** **amounts.** You do not need to actually weigh the food. You can estimate the number of teaspoons, cups *etc.*, and then state whether the spoons or cups are level, rounded or heaped.
2. **Use** **the** **weights** **marked** **on** **packages.** All packaged foods will have the weight marked on the packaging and this can be quoted as a reference, e.g., half a 425 g can of baked beans.
3. Please indicate the size of slices of bread, *i.e.*, sandwich, medium or toast.

PHYSICAL ACTIVITY LEVELS

An estimate of your physical activity on the days that you are recording your food and drink intake is also required. Instruction for this are listed on the physical activity record sheets.

Once you have completed the diet and physical activity survey, please bring it along with you to our study centre on your visit 1.

Five diet and physical activity record sheets have been supplied should you require an extra.

A code number will be written by us on the top left hand corner for our reference.

**DIETARY INTAKE**

| DATE |
| --- |
| Breakfast |
| Morning Snack |
| Lunch |
| Afternoon Snack |
| Dinner |
| Supper Snack |
| Dietary supplements |

**DIETARY INTAKE**

| DATE |
| --- |
| Breakfast |
| Morning Snack |
| Lunch |
| Afternoon Snack |
| Dinner |
| Supper Snack |
| Dietary supplements |

**DIETARY INTAKE**

| DATE |
| --- |
| Breakfast |
| Morning Snack |
| Lunch |
| Afternoon Snack |
| Dinner |
| Supper Snack |
| Dietary supplements |

**DIETARY INTAKE**

| DATE |
| --- |
| Breakfast |
| Morning Snack |
| Lunch |
| Afternoon Snack |
| Dinner |
| Supper Snack |
| Dietary supplements |

**DIETARY INTAKE**

| DATE |
| --- |
| Breakfast |
| Morning Snack |
| Lunch |
| Afternoon Snack |
| Dinner |
| Supper Snack |
| Dietary supplements |

PHYSICAL ACTVITY RECORD

A: Please indicate the description that best describes your lifestyle by circling a number the lifestyle description table below.

| **Description** **of** **Lifestyle** |
| --- |
| **1.** At rest, exclusively sedentary or lying.  (Chair bound or bed bound) |
| **2.** Exclusively sedentary activity/seated  work with little or no strenuous activity |
| **3.** Sedentary activity/seated work with  some requirement for occasional walking  and standing but little or no strenuous  leisure activity |
| **4.** Predominantly standing or  walking work |
| **5.** Heavy occupational work or highly  active leisure |
| **6.** Significant amounts of sport or strenuous leisure activity in addition to 2,3,4 above |
